# Kank Is an EB1 Interacting Protein that Localises to Muscle-Tendon Attachment Sites in *Drosophila*

**DOI:** 10.1371/journal.pone.0106112

**Published:** 2014-09-09

**Authors:** Sara M. R. Clohisey, Nikola S. Dzhindzhev, Hiroyuki Ohkura

**Affiliations:** The Wellcome Trust Centre for Cell Biology, School of Biological Sciences, The University of Edinburgh, Edinburgh, United Kingdom; Cardiff University, United Kingdom

## Abstract

Little is known about how microtubules are regulated in different cell types during development. EB1 plays a central role in the regulation of microtubule plus ends. It directly binds to microtubule plus ends and recruits proteins which regulate microtubule dynamics and behaviour. We report the identification of Kank, the sole *Drosophila* orthologue of human Kank proteins, as an EB1 interactor that predominantly localises to embryonic attachment sites between muscle and tendon cells. Human Kank1 was identified as a tumour suppressor and has documented roles in actin regulation and cell polarity in cultured mammalian cells. We found that *Drosophila* Kank binds EB1 directly and this interaction is essential for Kank localisation to microtubule plus ends in cultured cells. Kank protein is expressed throughout fly development and increases during embryogenesis. In late embryos, it accumulates to sites of attachment between muscle and epidermal cells. A *kank* deletion mutant was generated. We found that the mutant is viable and fertile without noticeable defects. Further analysis showed that Kank is dispensable for muscle function in larvae. This is in sharp contrast to *C. elegans* in which the Kank orthologue VAB-19 is required for development by stabilising attachment structures between muscle and epidermal cells.

## Introduction

Microtubules are dynamic polar polymers that perform vital functions in eukaryotic cells. The microtubule network constantly alters its dynamics and organisation according to the requirements of the cell, for example forming the spindle during cell division and forming a network which structurally supports the cell. These changes are mainly regulated by proteins that interact with microtubules, collectively called microtubule-associated proteins (MAPs) [1]. MAPs are a wide range of proteins with diverse structures and functions. So far, it has been a challenge to identify the molecular basis of tissue specific microtubule dynamics and organisation during development.

A subset of MAPs associate with growing ends of microtubules. EB1 is highly conserved from humans to yeast and has been shown to be necessary for dynamics at plus-ends [2], [3]. This protein was originally identified as a binding partner of APC (adenomatous polyposis coli) [4] and was later shown to track growing microtubule plus ends in cells [5]. It has been shown that EB1 plays a central role in regulation at microtubule plus ends [6], as it can bind microtubule plus ends directly [7] and can recruit various proteins with a range of structures and functions. Two sequence motifs have been identified which mediate the interaction with EB1, namely the CAP-Gly domain and the SxIP motif [8]–[10].

Although many studies on EB1 have been carried out in cultured cells, understanding of the roles and actions of EB1 are limited in the context of the whole organism. EB1 may regulate microtubule plus end behaviour differently in different cell types, as it recruits cell type specific effectors to microtubule plus ends. Systematic identification of EB1 interacting proteins has been carried out using mass-spectrometry [10], [11], but the choice of starting materials limits which proteins can be identified. Identification of EB1-interacting proteins differentially expressed in different tissues, such as muscle and the epidermis, will be a key step to determining how microtubule ends are regulated in different cell types.

In this study we identify the sole *Drosophila* orthologue of human Kank1–4 as an EB1-interacting protein, found to localise predominantly at sites of muscle-tendon attachment. The conserved protein Kank1 was identified as a human tumour suppressor [12], though exactly how it suppreses tumour growth remains unclear. So far, investigation of the mammalian Kank proteins has been carried out primarily in cell culture and they have been shown to have roles in inhibition of actin nucleation, actin organisation [13], [14], cell polarity [15] and cell growth [16]. A study in *C. elegans* shows that the sole Kank orthologue, VAB-19, localises to epidermal attachment structures between muscle and epidermal cells in developing nematode embryos, and later at circumferential bands that cover the length of the worm [17]. Disruption of VAB-19 during development is lethal, likely resulting from the detachment of muscles from the epidermis during elongation.

*Drosophila*, like *C. elegans*, contains a single Kank orthologue, CG10249, which we will call Kank in this report. Kank has not been studied in detail, however recent studies have indicated a role for Kank in neurogenesis and development [18]–[20].

Here we report on the identification of *Drosophila* Kank as a novel EB1-interacting protein with a specific localisation during embryogenesis. We demonstrate that Kank interacts with EB1 in S2 cells and requires EB1 for localisation to microtubule ends. Furthermore, we show that this interaction with EB1 is through an SxIP motif present in Kank. Additionally, we establish that Kank is expressed at most stages of the *Drosophila* lifecycle and its expression increases during embryonic development. Complete deletion of Kank coding sequence from the genome shows that Kank is dispensable for viability and fertility. We observe that Kank specifically localises to sites of attachment between muscle and tendon cells in embryos, suggesting a potential role in muscle-tendon attachment.

## Results

### Kank is associated with microtubule plus ends in an EB1 dependent manner

**Figure 1:**
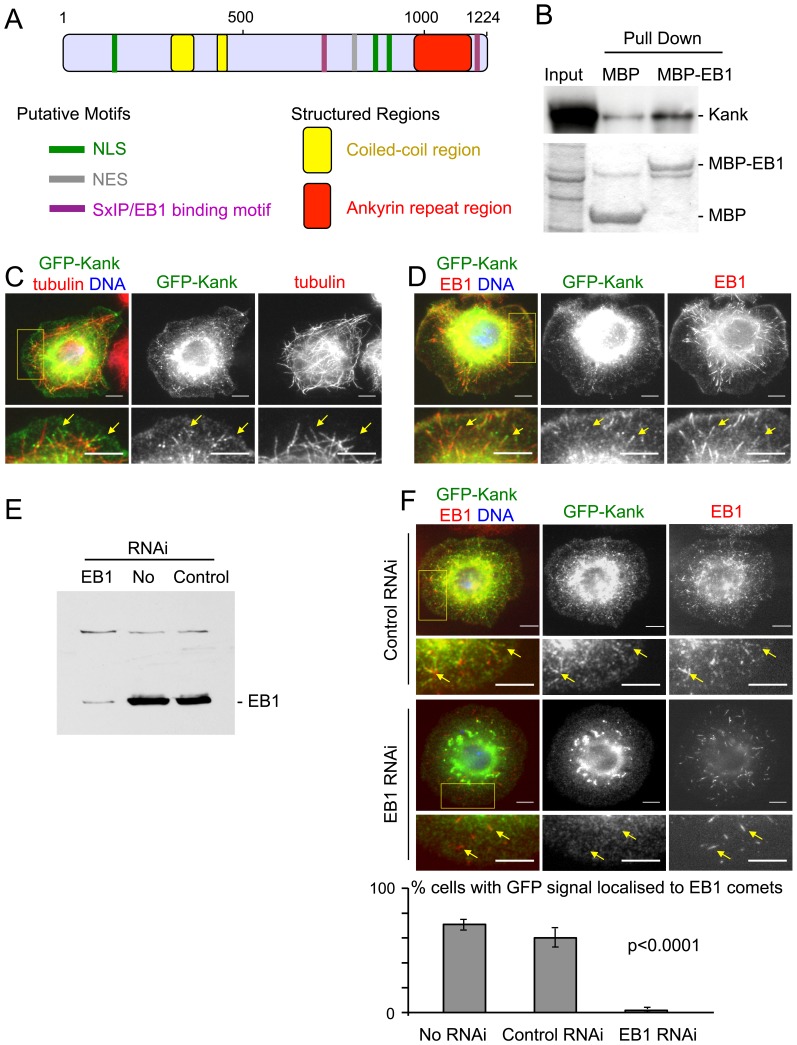
The conserved protein Kank directly interacts with EB1 *in vitro* and requires EB1 to localise to microtubule ends in cultured S2 cells. (A) *Drosophila* Kank contains the conserved structural elements of Kank family proteins. *Drosophila* Kank also contains putative, conserved motifs including nuclear localisation signals (NLS in green), nuclear export signals (NES in grey) and SxIP/EB1 binding motifs (in purple). (B) Kank produced by *in vitro* transcription/translation in the presence of ^35^S-methionine was successfully pulled down by bacterially expressed MBP-EB1 but not by MBP alone. Autoradiograph and protein staining are shown at the top and bottom panels respectively. The input is equivalent to 30% of the pull downs. (C) Cultured S2 cells were transfected with GFP-Kank and immunostained for GFP and α-tubulin. The GFP signal localises to the cytoplasm, largely to microtubule ends (yellow arrows). Minor localisation was also observed at the cell periphery. (D) S2 cells transfected with GFP-Kank were immunostained for GFP and EB1. The GFP signal largely co-localised with EB1 comets (yellow arrows). (E) Immunoblotting confirmed the reduction of EB1 by RNAi. (F) Kank requires EB1 to localise to microtubule ends. RNAi of EB1 in S2 cells led to delocalisation of GFP-Kank from microtubule ends, compared to control β-lactamase RNAi cells. The percentage of cells with GFP-Kank signal localised to the majority of visible EB1 comets (>50%, estimate) was counted. The GFP-Kank signal at EB1 comets was significantly reduced in EB1 RNAi cells compared to control RNAi cells and control cells without RNAi. Error bars show the standard error of the mean. For all microscopy images yellow boxes are areas magnified in images shown below and co-localisation is indicated by yellow arrows. Scale bars = 5 µm.

To understand how microtubule plus ends are regulated during development, we have carried out *in vitro* expression screening for EB1 interacting proteins. In brief, cDNAs from an annotated cDNA collection (*Drosophila* Gold collection) were transcribed and translated *in vitro*. The products were pulled down using bacterially produced MBP-EB1 or MBP alone to identify proteins which specifically interact with EB1. As these cDNAs were isolated from various tissues and developmental stages, we hoped that this method would identify EB1 interacting proteins even if they are only expressed in certain cell types and developmental stages. This is in contrast to mass spectrometry methods which only identify proteins expressed in specific starting cells. The gene product of *CG10249* (Figure 1A) was identified as a protein which can directly bind EB1 *in vitro* (Figure 1B). CG10249 is conserved among higher eukaryotes and highly homologous to human Kank1-4. This is the sole member of the Kank family of proteins in *Drosophila melanogaster*, and therefore called Kank here.

To determine whether Kank can localise to microtubule plus ends, GFP fused full-length Kank (GFP-Kank) was expressed in *Drosophila* cultured S2 cells. S2 cells were transfected with a plasmid expressing GFP-Kank under the *actin5C* promoter and immunostained for GFP and α-tubulin (Figure 1C). In interphase cells, GFP-Kank showed diffuse cytoplasmic localisation in addition to its primary localisation with the ends of microtubules. The intensity and length of the GFP signal at microtubule ends was variable. As Kank interacts with EB1 *in vitro*, localisation of GFP-Kank relative to EB1 in S2 cells was investigated by co-immunostaining (Figure 1D). In most cells (76%), GFP-Kank co-localised with the majority of EB1 comets.

EB1 has been shown to recruit proteins to the plus ends of microtubules [6], [9], [21]. To test whether EB1 is required for the microtubule plus end localisation of GFP-Kank, we depleted EB1 from S2 cells by RNA interference (RNAi) and transfected these cells with a plasmid expressing GFP-Kank. A western blot confirmed a reduction of EB1 protein in the cells (Figure 1E), although a small amount of EB1 was observed as comets at microtubule plus ends (Figure 1F). Immunostaining of GFP and tubulin showed that the localisation of GFP-Kank to microtubule plus ends was greatly reduced in S2 cells after EB1 RNAi. This reduction was not observed in control RNAi cells (Figure 1F). This demonstrated that the localisation of GFP-Kank at microtubule plus ends is dependent on EB1.

### Kank contains an SxIP motif that is required for localisation to microtubule plus ends

**Figure 2:**
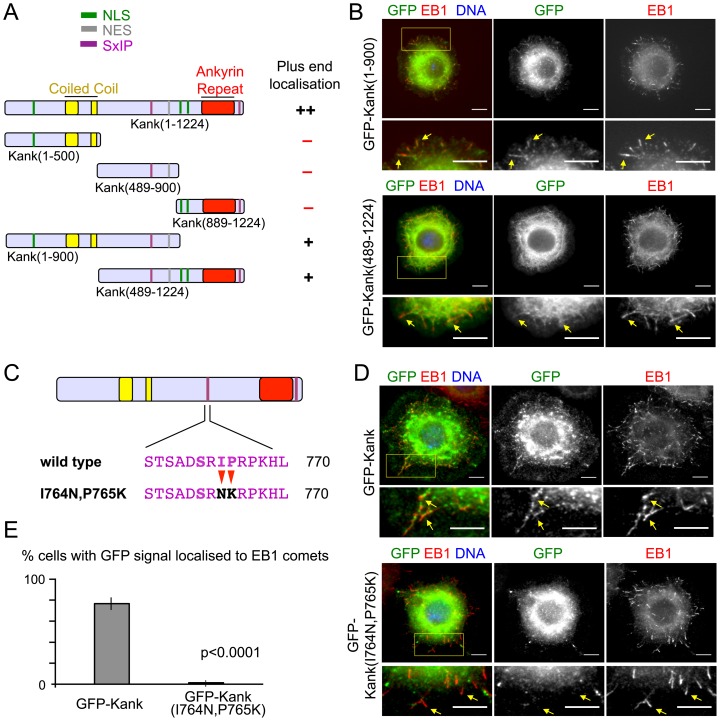
Kank/EB1 binding is via an EB1 binding, SxIP, motif in the middle region of Kank. (A) A summary of Kank truncations and their microtubule plus end localisation. ++ (strong localisation), + (weak localisation), - (no localisation). (B) Kank truncations which contain the middle region of Kank can co-localise with EB1 in S2 cells. S2 cells transfected with GFP-fused truncations were co-stained for GFP, EB1 and DNA. Co-localisation was observed (yellow arrows). (C) The SxIP motif in Kank was mutated to SxNK. (D) S2 cells transfected with GFP-Kank(I764N,P765K) were co-stained for GFP and EB1. Mutation of the SxIP motif in the middle region of Kank abolished co-localisation of Kank with EB1 (yellow arrows). (E) Mutation of the SxIP motif significantly decreased the percentage of cells with GFP signal localised to the majority of the observed EB1 comets. Significance was determined by Fishers exact chi squared test. Error bars show the standard error of the mean. For all microscopy images yellow boxes are areas magnified in images shown below and co-localisation is indicated by yellow arrows. Scale bars = 5 µm.

To identify the cis-element of Kank protein responsible for its localisation, various regions of Kank fused to GFP were transiently expressed in S2 cells under the *actin5C* promoter, and their localisation was determined by immunostaining (Figure 2A). Kank proteins lacking the C-terminal or the N-terminal region, Kank(1–900) and Kank(489–1224), show localisation to microtubule plus ends similar to, but weaker than, full-length Kank (Figure 2B and Figure S1). Smaller regions of Kank, Kank(1–500), Kank(489–900) and Kank(889–1224), did not show microtubule plus end localisation (Figure S2). Many proteins that EB1 recruits to microtubule plus ends contain SxIP motifs that mediate direct interaction with EB1 [9]. A sequence that matches to the consensus SxIP motif is located in the middle region shared by two Kank truncations which localise to microtubule plus ends. To determine if this SxIP motif was responsible for microtubule plus end localisation, the SxIP sequence was mutated to SxNK creating Kank(I764N,P765K) (Figure 2C). Transient expression in S2 cells showed that GFP-Kank(I764N,P765K) was diffuse in the cytoplasm with very little signal co-localised with EB1 to microtubule ends (Figure 2D). The frequency of cells with GFP signal at the majority of the EB1 comets was greatly reduced in GFP-Kank(I764N,P765K) (1.5%) compared to cells expressing GFP-Kank (76.5%) (Figure 2E). These results show that the SxIP motif in the middle region of Kank is essential for Kank localisation to EB1 and thus microtubule plus ends.

### Kank can shuttle between the nucleus and the cytoplasm

**Figure 3:**
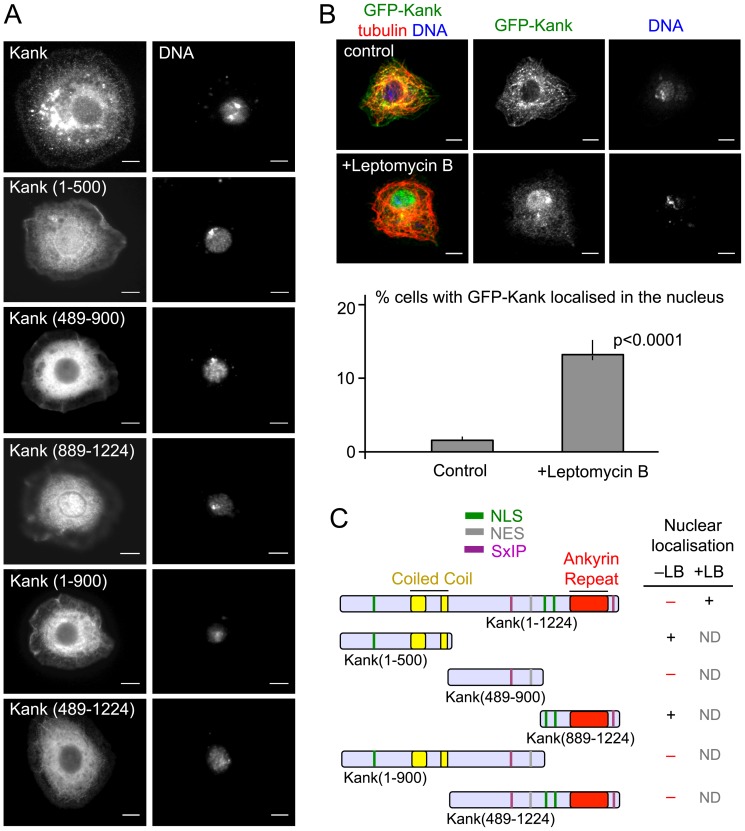
Kank can localise transiently to the nucleus of S2 cells. (A) Kank(1–500) and Kank(889–1224) exhibit nuclear localisation, while truncations of Kank which contain the middle region do not. S2 cells were transfected with GFP-fused Kank truncations. Cells were then co-stained for GFP, α-tubulin and DNA. (B) Kank shuttles between the nucleus and the cytoplasm in some cells. S2 cells transfected with GFP-Kank were incubated with media containing leptomycin B for 3–3.5 hours, to inhibit nuclear export. Control cells were incubated with media containing methanol, the solvent for leptomycin B. Nuclear localisation was observed more frequently in leptomycin B treated cells than control cells. Significance was determined by Fishers exact chi squared test. Error bars show the 95% confidence interval. (C) A summary of Kank truncations and their nuclear localisation. + and – indicate the presence and absence of the nulcear localisation with (+LB) or without (–LB) Leptomycin B. ND (Not done). Scale bars = 5 µm.

Interestingly, Kank(1–500) and Kank(889–1224) both exhibit strong nuclear localisation in most transfected cells (Figure 3A,C). GFP-Kank(489-900) shows a weak nuclear localisation in only a minority of cells. Nuclear localisation was not observed for full-length Kank, Kank(1–900) or Kank(489–1224). This may be because Kank has both nuclear localisation signals and a nuclear export signals, or Kank has a cryptic nuclear localisation signal that is usually non-functional. Analysis of the Kank sequence indicates 3 putative NLSs, one within Kank(1–500) and two within Kank(889–1224) (Figure 1A). A putative NES was identified within Kank(489–900). To determine if full-length Kank could localise transiently to the nucleus, nuclear export was inhibited in cells expressing GFP-Kank by addition of leptomycin B (Figure 3B). Leptomycin B inhibits nuclear export by the active transporter CRM1 leading to an accumulation of NLS containing proteins in the nucleus [22]. Accumulation of GFP-Kank in the nucleus was observed in some cells (14%) 3 hours after the addition of leptomycin B, in comparison to cells without the drug (<2%) (Figure 3B). This suggests that Kank shuttles between the cytoplasm and the nucleus.

### Kank is expressed throughout fly development but is dispensable for viability and fertility

**Figure 4:**
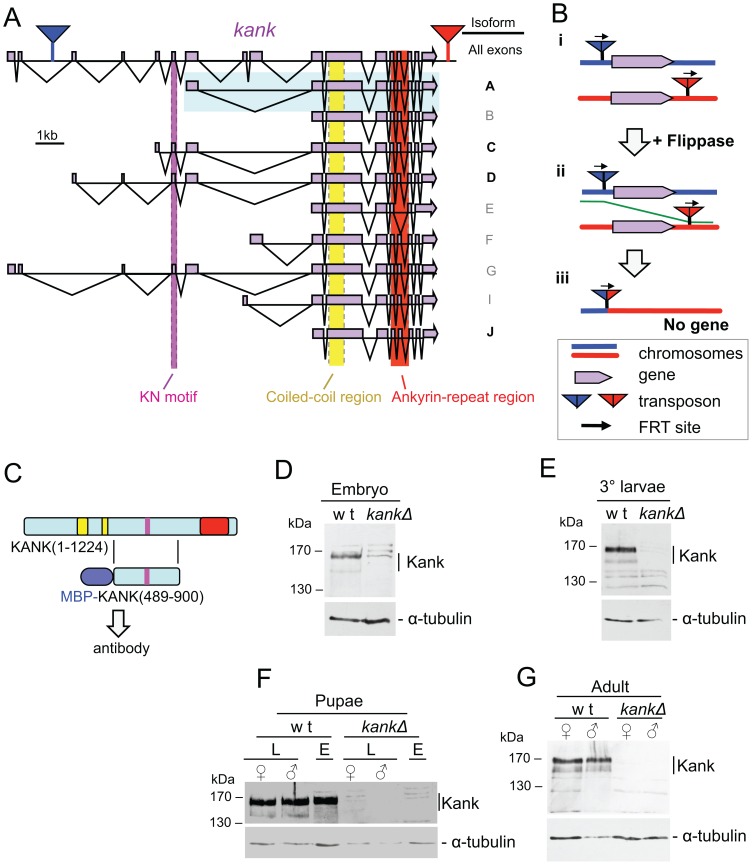
Kank is expressed throughout development but is dispensable for viability and fertility. (A) *kank* (*CG10249*) is a ∼27 kb gene found at 51D2 on chromosome arm 2R. Putative isoforms are shown. Those in black are likely to be expressed while those in grey are less likely to be expressed based on ModEncode data. The cDNA clone GH03482 that we used in our analysis represents isoform A of *kank* (highlighted in blue). This isoform lacks the KN motif found in other Kank proteins (shown in purple). (B) Kank was deleted using transposons containing *FRT* sites. Firstly, the appropriate two transposons flanking the Kank coding sequence were introduced in trans positions on homologous chromosomes (i). A flippase was induced to promote recombination between the *FRT* sites (ii) and generated a deletion of the intervening sequence (iii) (C) The fragment of Kank(489–900) used for generating an antibody against the Kank protein. (D–G) The Kank antibody detected the endogenous protein in all lifecycle stages examined by immunoblotting in wild type but not in *Kank* deletion mutants. Kank was detected in embryos 21–24 hrs after egg laying (D), in 3rd instar larvae (E), in male and female late pupae [ = L] and early pupae of undetermined gender [ = E] (F), and in both male and female adult flies (G).

To make a deletion of the *kank* gene, we used two chromosomes each carrying a transposon insertion containing a *FRT* site [23] which are located at either side of the coding sequence of *kank*. Flippase was expressed in transheterozygotes carrying both transposons to induce recombination between the two *FRT* sites (Figure 4A,B) and recombination events which produce the deletion of the genomic region between the two *FRT*s were selected for. This resulted in 3 strains in which the entire Kank-coding sequence was deleted (*kankΔ1*, *kankΔ2*, *kankΔ3*). Deletion of expected regions was confirmed by PCR (Figure S3), and the absence of the Kank protein was confirmed by western blotting (Figure 4D–G). Unexpectedly, unlike *C. elegans* in which mutation of the *kank* orthologue, *vab-19*, is lethal, the *kank* deletion was homozygous viable and fertile, therefore a homozygous stock could be established. Viability was confirmed at 18°C and 29°C.

To detect the Kank protein in cells or cell extracts, we have generated antibodies which recognise the Kank protein. To establish when Kank is expressed during development, total protein samples from 21–24 hours old embryos, third instar larvae, early pupae, late pupae and adult flies were prepared. All samples were analysed by western blotting using an affinity purified Kank antibody against Kank(489–900) (Figure 4C–G). A band around 160 kDa, which may represent a set of proteins with slightly different mobility, was detected in all developmental stages in wild type. A weak band around 140 kDa is also visible, possibly representing either a degradation product or an alternative isoform of Kank. These bands were not present in protein samples prepared from the *kank* deletion mutant at the equivalent stages, confirming that these bands correspond to the Kank protein. These results indicate that Kank is expressed throughout the lifecycle of the fly.

### Kank localises to muscle-tendon attachment sites in *Drosophila* embryos

**Figure 5:**
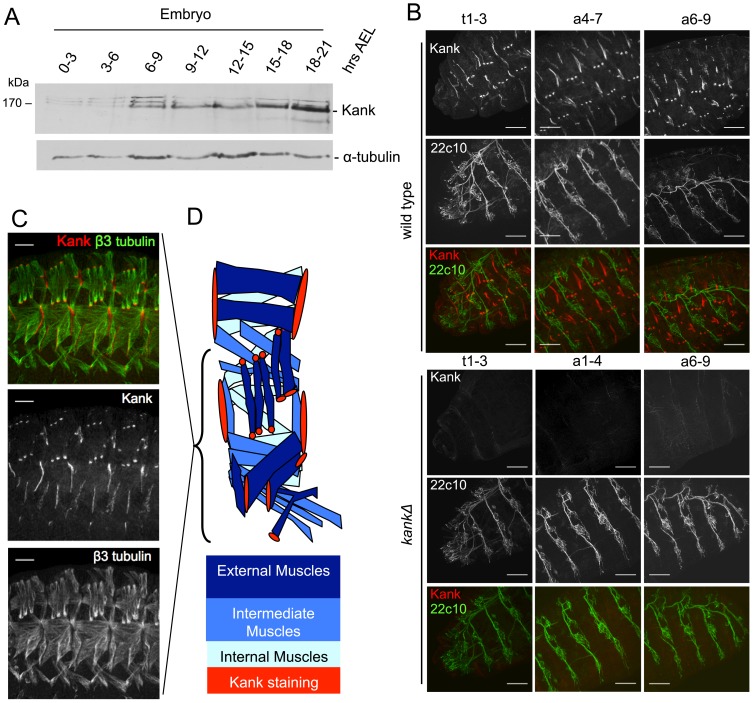
Kank localises to muscle-tendon attachment sites in late stage *Drosophila* embryos. (A) The ∼160 kDa Kank band was detected in embryos from 3–6 hours after egg laying (AEL). The amount of Kank detected by immunoblotting was observed to increase during embryonic development. The ∼140 kDa band becomes apparent 15–18 hours AEL. (B) An antibody against Kank(489–900) stained a distinct pattern in stage 16/17 embryos. This staining was not observed in *kank* deletion mutants. The 22c10 antibody, which highlights neurons, was used to orient embryos. (C) β3-tubulin staining reveals the structure of microtubules in somatic muscle cells. Co-staining with the Kank antibody showed that Kank localises at sites of muscle attachment to the epidermis. (D) A schematic of *Drosophila* embryonic somatic musculature with sites of Kank staining indicated. Scale bars = 25 µm.

To determine if Kank protein levels change throughout embryogenesis, embryos were collected for 3 hours and aged for various lengths at 25°C. Protein samples from these embryos were analysed by western blotting using the Kank antibody (Figure 5A). The Kank bands were not visible in 0–3 hour old embryos, suggesting little or no maternal contribution. The 160 kDa band was visible from 3 hours after egg laying (AEL) and increased in intensity as the embryos age, until roughly 15–18 hours which is equivalent to the stages 16/17 of embryo development (Figure 5A). The smaller band becomes visible 15–18 hours AEL. These bands are absent in the *kank* deletion mutant. This expression pattern during development indicates that expression of Kank may be temporally regulated during embryogenesis.

To determine the localisation of Kank in embryos, wild-type embryos of various ages were immunostained with our Kank antibody and with the monoclonal antibody 22c10 which recognises the Map1b-like protein Futsch and highlights neurons [24]. In embryos at stages 16/17 or later, Kank showed very clear, distinct localisation (Figure 5B). Each hemi segment (half segment) distinctly displays eight spots of Kank signal, arranged as four spots in a line along the ventral-lateral axis and four parallel spots in a line along the dorsal-lateral axis, the posterior most spots are shifted dorsally. These signals were not observed in the *kank* deletion embryos (Figure 5B), confirming that they represent Kank localisation.

The position of the signal appears to coincide with sites at which muscle and tendon cells attach [25]. Co-staining of Kank with β-3 tubulin, which is preferentially expressed in muscle cells, confirmed that Kank is localised at muscle-tendon attachment sites (Figure 5C).

**Figure 6:**
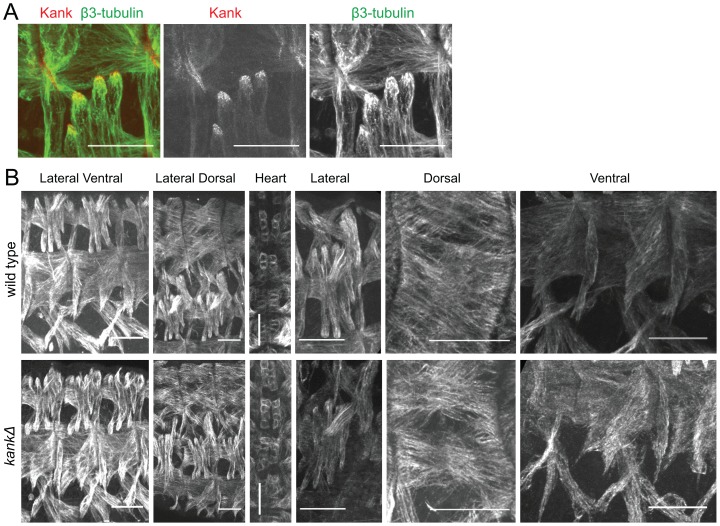
Deletion of *kank* did not affect muscle cell morphology or microtubule organisation. (A) Closer examination of the lateral transverse muscle shows Kank staining near sites where microtubule ends are attached to the periphery of muscle cells. (B) No clear differences were seen in the overall organisation of somatic musculature, or in the organisation of their microtubules, between wild type and *kankΔ* as imaged with β3-tubulin staining. Scale bars = 25 µm.

Closer observation revealed that the Kank signal overlaps with microtubules and is strongly concentrated near sites where microtubule ends are attached to the periphery of muscle cells (Figure 6A). It is unclear whether the Kank signal is in muscle or tendon cells. This localisation was confirmed when embryos were co-stained for Kank and actin (Figure S4). As Kank protein localises at sites of muscle attachment to epidermal cells, the muscle morphology and microtubule organisation was examined using the β3-tubulin antibody. No clear differences were observed between wild type and the Kank deletion mutant (Figure 6B). Additionally, we tested muscle and sensory function in larvae using a wide range of assays (Figure S5) and surprisingly did not find any significant differences between *kankΔ* mutant and wild-type larvae, despite the specific localisation of the protein.

## Discussion

EB1 is a key protein which regulates microtubule plus ends through the recruitment of other proteins [6], [21]. In this study, we have identified Kank as an interacting protein of EB1 using *in vitro* expression cloning. We have shown that Kank localises to microtubule plus ends in an EB1-dependent manner in culture cells. Kank is predominantly localised to the attachment region of muscle and epidermal cells in late embryos. Flies completely lacking Kank are viable and fertile, and show no defects in muscle or sensory function in our assays.

Here we used *in vitro* expression cloning to identify EB1 interacting proteins. As the annotated cDNA collection used here has been derived from various developmental stages and tissues and each gene is represented only once, we rationalised that this approach would identify EB1-interacting proteins in an unbiased way regardless of the expression levels in particular cell types. Kank, the sole *Drosophila* orthologue of the human tumour suppressor Kank1, was identified. This data indicates that Kank may have a role in linking actin and microtubule regulation during development.

Interaction between EB1 and Kank is shown by *in vitro* pull down experiment and colocalisation in S2 cells. RNAi of EB1 in GFP-Kank transfected S2 cells demonstrates that EB1 is required for microtubule plus end localisation of Kank. We further showed that an EB1 interaction motif (SxIP motif) of Kank is essential for its localisation to microtubule plus ends in S2 cells. This sequence is conserved among *Drosophila* and consensus sequences are present in human Kank1 and Kank4. A recent proteomic study identified Kank2 as a putative EB1 binding protein, though this was not confirmed by other methods [10]. It would be of future interest to see whether the mammalian and *C. elegans* Kank orthologues associate with microtubules or with EB1.

We have also demonstrated that Kank, like human Kank1, has the capacity to localise to the nucleus and likely does so transiently. Using truncations, we identified the N-terminus and the C-terminus as regions of Kank which can localise to the nucleus while truncations of Kank which contain the middle region do not. Treatment of S2 cells with leptomycin-B has shown that GFP-Kank shuttles between the cytoplasm and the nucleus, at least in a minority of cells. Human Kank1 has been shown to accompany the transport of β-catenin from the cytoplasm to the nucleus in some cell types [26] and it is possible this function is conserved in *Drosophila*.

Deletion of the *kank* coding region reveals that Kank is dispensable for viability and fertility in *Drosophila*. This is in contrast with what was observed in *C. elegans*, where disruption of VAB-19 expression was fatal. Additionally, we found that, despite the developmental disorders observed in humans [27], [28], *Drosophila kankΔ* larvae do not display any motility or muscular defects.

Kank is present throughout the *Drosophila* life cycle and its expression increases during embryogenesis. At late embryonic stages, the pattern of Kank localisation coincides with muscle-tendon attachment sites in developing embryos. How this localisation of Kank relates to the function of the protein has yet to be determined. In *C. elegans*, GFP-VAB-19 localises to muscle-epidermal attachment structures, consistent with the localisation observed for Kank and is essential to maintain myotactin, VAB-10A and intermediate filaments at attachment sites [17]. *Drosophila* does not contain any cytoplasmic intermediate filaments [29], often substituting arrays of microtubules in their place [30]. The *Drosophila* homologue of myotactin, *sidekick*, functions in cell adhesion at synapses in the retina [31] but its function in muscles has not been examined. Shot, the *Drosophila* homologue of VAB-10A, has been demonstrated to be required in tendon cells for muscle-tendon junction formation [32]. It is required for the localisation of EB1 and actin-microtubule interaction in these cells [33], [34]. Interestingly, Shot also displays a similar localisation to Kank in the late stages of embryogenesis [25]. Specifically Shot localises to the area enriched in microtubule ends in tendon cells [34]. Given the role of mammalian Kank proteins in actin regulation, it would be of a future interest to test functional interactions between Kank and Shot in *Drosophila*. In humans it has been shown that an isoform of human Kank1 has higher expression in adult skeletal muscle, liver, heart, kidney and tissues than in other tissues, though no studies of embryonic tissues exist [35].

We have generated a complete deletion of Kank coding sequence. Flies lacking Kank are viable and fertile, and our wide range of assays did not identify defects in muscle activities in a third instar larvae. It is possible that our assays were not sensitive enough to detect the specific function of Kank. Considering the localisation of Kank in *Drosophila* embryos, it may have a specific function in interaction between muscle and epidermal cells. Alternatively, the Kank function may be masked by a redundancy with other proteins, for example Shot or other EB1 interacting proteins. It will be interesting to further uncover such muscle specific EB1 interacting proteins and determine the intricate complexes formed at the site of muscle-tendon attachment.

## Materials and Methods

### Identification of Kank/CG10249 as an EB1 interactor

Kank was identified by *Drosophila in vitro* expression cloning. A pool of 24 cDNAs from an annotated collection of *Drosophila* cDNAs was transcribed and translated *in vitro* in the presence of ^35^S- methionine (Easytag, Perkin Elmer) using the T7 TnT Quick Coupled system (Promega). Each translated product was split into two and incubated in DIVEC buffer (50 mM Hepes pH 7.6, 1 mM MgCl_2_, 1 mM EGTA, 200 mM NaCl, 0.5% Triton-X100) for 60 minutes with amylose resin (New England Biolabs) coupled with bacterially-produced MBP or MBP-EB1. After extensive washing in DIVEC buffer, the beads were boiled with the sample buffer and run on an SDS gel. Dried gels were exposed to X-ray film (Hyperfilm, GE Healthcare). cDNA pools which gave bands specific for MBP-EB1 pull down were further studied by testing sub-pools until a single responsible cDNA was identified.

### Molecular and Protein Techniques

Standard DNA and protein techniques were used throughout [36]. The *kank* coding region (GH03482) was introduced first into the Gateway entry vector pDONR221, and then into destination vector, pAGW to generate a plasmid for expression of Kank fused to GFP at the N-terminus under the *actin5C* promoter. Regions of *kank* were amplified using the appropriate primers which facilitated the addition of *attB* sites, allowing introduction of the *Kank* regions into the appropriate vectors. To mutate the EB1 binding motif in Kank, two single nucleotide substitutions were introduced. To mutate amino acid 764 (I→N) nucleotide 2339 of GH03482 was mutated (T→A) using the following primer pair (forward/reverse), CGGCGGACTCGAGAAATCCGCGACCCAAGC and GCTTGGGTCGCGGATTTCTCGAGTCCGCCG. To mutate amino acid 765 (P→K) nucleotides 2341 and 2342 of GH03482 were mutated (CC→AA) using the following primer pair (forward/reverse), CCGTCGGACTCGAGAAATAAGCGACCCAAGCACCTC and GAGGTGCTTGGGTCGCTTATTTCTCGAGTCCGACGG using Quick Change XLII site directed mutagenesis kit (Agilent), following manufacturer's instructions. Antibodies against Kank (1∶100) and mouse anti-α-tubulin 1∶1000 (DM1A, Sigma) were used for western blotting and detected by the ECL system (Amersham Biosciences) according to the manufacturer's protocols.

### Antibody Generation

Rabbit antibodies were raised to MBP-Kank(489–900) in the following way: Purified protein (250 µg) was injected at regular intervals. The final bleed from each antibody was used for antibody purification by a method using antigen immobilised on nitrocellulose membrane [37]. 20 µg of purified antigen was run on an SDS gel and transferred onto a nitrocellulose membrane. The membrane was stained with 1% Ponceau S in 1% acetic acid and the band of antigen excised. The band was washed and blocked and the antibody was then bound to the antigen by incubating the membrane in 100 µl of the final antiserum diluted 1∶10 in blocking solution overnight at 4°C. After extensive washes in washing buffer, the antibody was eluted by three, consecutive 30 second washes with 400 µl elution buffer (50 mM glycine-HCl pH2.3, 0.5 M NaCl, 0.5% Tween 20, 100 µg/µl BSA, 0.1% NaN_3_). All of the eluates were combined and immediately neutralised by adding Na_2_HPO_4_ solution to a final concentration of 50 mM. The affinity purified antibodies were tested for specificity and optimal dilution in immunoblotting and immunofluorescence experiments. Antibodies were stored at 4°C.

### Cell culture

*Drosophila* Schneider S2 cells were cultured. Transfections and RNA interference (RNAi) were performed according to published methods [38]. Plasmids were transfected using Effectene transfection reagent (Qiagen) following manual's instructions. Double-stranded RNA (dsRNA) corresponding to regions amplified by primer pairs (forward/reverse), CGACTCACTATAGGAAGAATGGCTGTAAACGTCTAC and CGACTCACTATAGGGAGATGCCCGTGCTGTTGGCAC for EB1 were used. dsRNA corresponding to *E. coli* beta-lactamase was used as a control.

### Cytological Analysis

S2 cells were plated on Concanavalin A coated coverslips for 2–3 hours and fixed with 90% methanol, 3% formaldehyde, 5 mM NaHCO_3_ pH9 at −80°C. Cells were stained with the following primary antibodies: rabbit anti-Kank(489–900) 1∶20, rabbit anti-EB1 1∶200, mouse anti-α-tubulin 1∶250 (DM1A, Sigma), rabbit anti-GFP 1∶500 (Molecular Probes), mouse anti-GFP 1∶500 (3E6, Molecular Probes). Cells were visualised with an Axioplan-2 fluorescence microscope (Zeiss) and images were recorded with an attached CCD camera (Hamamatsu), controlled by OpenLab 2.2.1 software (Perkin Elmer). Images were processed using ImageJ.

### *Drosophila* Techniques

Standard *Drosophila* techniques were used throughout [39] and *w^1118^* was used as wild type in this study. *kankΔ* mutants were generated by inducing, by heat shock, recombination between two *FRT*-containing transposons (*PBac{PB}c00393* and *PBac{WH}f01478*). Recombination resulted in a loss of the *w^+^* gene from both transposons upon deletion of the *kank* gene. Chromosomes which have lost the *w^+^* gene were selected for and tested over a deficiency uncovering the *kank* gene. No chromosomes lethal over the deficiency were isolated. Chromosomes were tested over the deficiency for the presence of the *kank* genomic region by PCR to confirm deletion of the region (Table S1).

### Embryo collection and staining

Embryos were collected on plates and aged appropriately. Embryos were then washed with deionised water and dechorionated in 2.5% chloros in water. For Kank/22c10 and Kank/Actin stained embryos: Embryos were washed thoroughly and transferred to a glass vial containing 1∶1 heptane: methanol. The vial was sealed and shaken for 1 minute after which the liquid was extracted with a pipette and replaced with fresh methanol. These embryos were then incubated at room temperature for over 4 hours. Rehydration was done by passaging the embryos through increased concentrations of PBS in methanol (20%, 40%, 60%, 80% 100%) for 10 minutes at a time and stained. For Kank/β3-tubulin stained embryos: embryos were stained according to previously published methods [40]. Antibody concentrations used were: 1∶500 rabbit anti-Kank(489–900), 1∶500 mouse 22c10 (Developmental Hybridoma Studies Bank), 1∶5000 guinea pig anti-β3 tubulin [41] and 1∶500 rabbit anti-β3 tubulin [42]. Embryos were then mounted in mounting medium (2.5% propyl gallate, 85% glycerol) and viewed on an LSM510 confocal microscope. Images were processed using ImageJ.

## Supporting Information

Figure S1 **Kank(1–889) and Kank(489–1224), co-localise with EB1.** S2 cells transfected with GFP-Kank(1–889) and GFP-Kank(489–1224) were co-stained for GFP and EB1. The number of cells with GFP localised to the majority of the EB1 comets (>50% estimate) was counted. For both truncations colocalisation of the GFP signal with EB1 was observed in the majority of observed cells. Error bars show the standard error of the mean.
(TIF)

Figure S2 **Kank(1–500), Kank(489–900) or Kank(889–1224) do not co-localise with EB1.** S2 cells were transfected with Kank(1–500), Kank(489–900) or Kank(889–1224) and co-stained for GFP and EB1. These truncations showed diffuse localisation within the cytoplasm. In addition, Kank(1–500) and Kank(889–1224) localised to the nucleus. GFP signal was observed at the cell periphery for Kank(1–500) and Kank(489–900). Yellow boxes are areas magnified in images shown below. Scale bars = 5 µm.
(TIF)

Figure S3 **Deletion of Kank was confirmed by PCR.** (A) PCR was carried out on genomic DNA of three *kank* deletion (*Δ1, Δ2, Δ3*) and two parental lines (*P[c00393]*, *P[f01478]*) using designated primers (Table S1).
(TIF)

Figure S4 **Kank localises to muscle tendon attachment sites.** As actin staining is quite ubiquitous, Z sections of actin stained embryos were examined to visualise cells which resembled those muscle cells indicated in the schematic. The localisation of the Kank signal is observed at the sites of muscle-tendon attachment. Coloured boxes show the similarities between actin staining and the somatic muscle schematic. Scale bar = 25 µm.
(TIF)

Figure S5 ***kankΔ*** **larvae do not exhibit any motility or sensory defects.** Unless otherwise stated, larvae examined were late 3rd instar (∼76 hours after hatching) and significance was determined by Student's t-test (A–C,E) or Fisher's exact chi-square test (D,F). Error bars show the standard error of the mean (A–C,E) or the 95% confidence interval (D,F). All assays were carried out at room temperature. (A) In the first assay the motility of larvae was examined (adapted from [43]) The number of gridlines passed by individual larva in 60 seconds was counted. The number of gridlines crossed by the *kankΔ* larvae was similar to that crossed by the wild type control (p>0.05). (B) In the second assay the overall coordination and motility of larvae was then examined by counting the number of full body motile waves (peristaltic waves) carried out by larvae in one minute (adapted from [44]). The peristaltic waves travelled the entire length of the larva in a coordinated fashion in both wild type and the mutant. The frequency of peristaltic waves was not significantly different between the *kankΔ* and wild type (p>0.05). (C) In the third assay, larvae were rolled from their ventral to their dorsal side while on an agarose plate (adapted from [45]). The time taken for them to right themselves was measured, with a maximum of 2 minutes allowed. The time taken by *kankΔ* and wild type was similar (∼30 seconds; p>0.05). (D) The fourth assay determined if larvae maintained burrowing ability. Foraging third instar larva were placed on top of food in a bottle which was then placed in the dark for 2 hours (adapted from [46]). After this time, the number of larvae remaining on the food was counted. No significant difference between the *kankΔ* and the wild-type larvae were observed (p>0.05). (E) Larvae were manually stimulated to elicit a nociceptive response (adapted from [47]). Third instar larvae was prodded by a blunt instrument at their abdominal segments and evading action was observed. No significant difference was found between *kankΔ* and wild type (p>0.05). The number of stimulations required to elicit a response was similar between strains. (F) *kankΔ* larvae have a wild type reaction to light (adapted from [48]). Foraging 3^rd^ instar larvae were placed on the midline of a plate with food. Half the plate was covered with aluminium foil and the plate was placed under a strong lamp in an otherwise dark room. Larvae were allowed to wander for 45–60 minutes after which the number on each side of the plate was counted. *kankΔ* larvae show an aversion to light equal to that of wild type.
(TIF)

Table S1 **Sequences of primers used to check for** ***kank*** **deletion in genomic DNA.**
(DOC)
