# Correction: Kank Is an EB1 Interacting Protein that Localises to Muscle-Tendon Attachment Sites in *Drosophila*

**DOI:** 10.1371/journal.pone.0114770

**Published:** 2014-12-01

**Authors:** The PLOS ONE Staff

The publisher apologizes for the following errors that were introduced during production.

Sara M. R. Clohisey should not have been designated as the corresponding author. Hiroyuki Ohkura (h.ohkura@ed.ac.uk) is the corresponding author.

There are errors in the current addresses for Sara M. R. Clohisey and Hiroyuki Ohkura. Sara M. R. Clohisey's current address is The Roslin Institute, University of Edinburgh, Easter Bush, Edinburgh, United Kingdom. Hiroyuki Ohkura is not affiliated with The Roslin Institute.

## References

[1] Clohisey SMR, Dzhindzhev NS, Ohkura H (2014) Kank Is an EB1 Interacting Protein that Localises to Muscle-Tendon Attachment Sites in *Drosophila* . PLoS ONE 9(9): e106112 doi:10.1371/journal.pone.0106112 25203404 10.1371/journal.pone.0106112 PMC4159139

