# Supplementary figures and images for: Kank Is an EB1 Interacting Protein that Localises to Muscle-Tendon Attachment Sites in Drosophila
Source: PLoS One. 2014 Sep 9;9(9):e106112. doi: 10.1371/journal.pone.0106112 (PMC4159139; doi:10.1371/journal.pone.0106112)

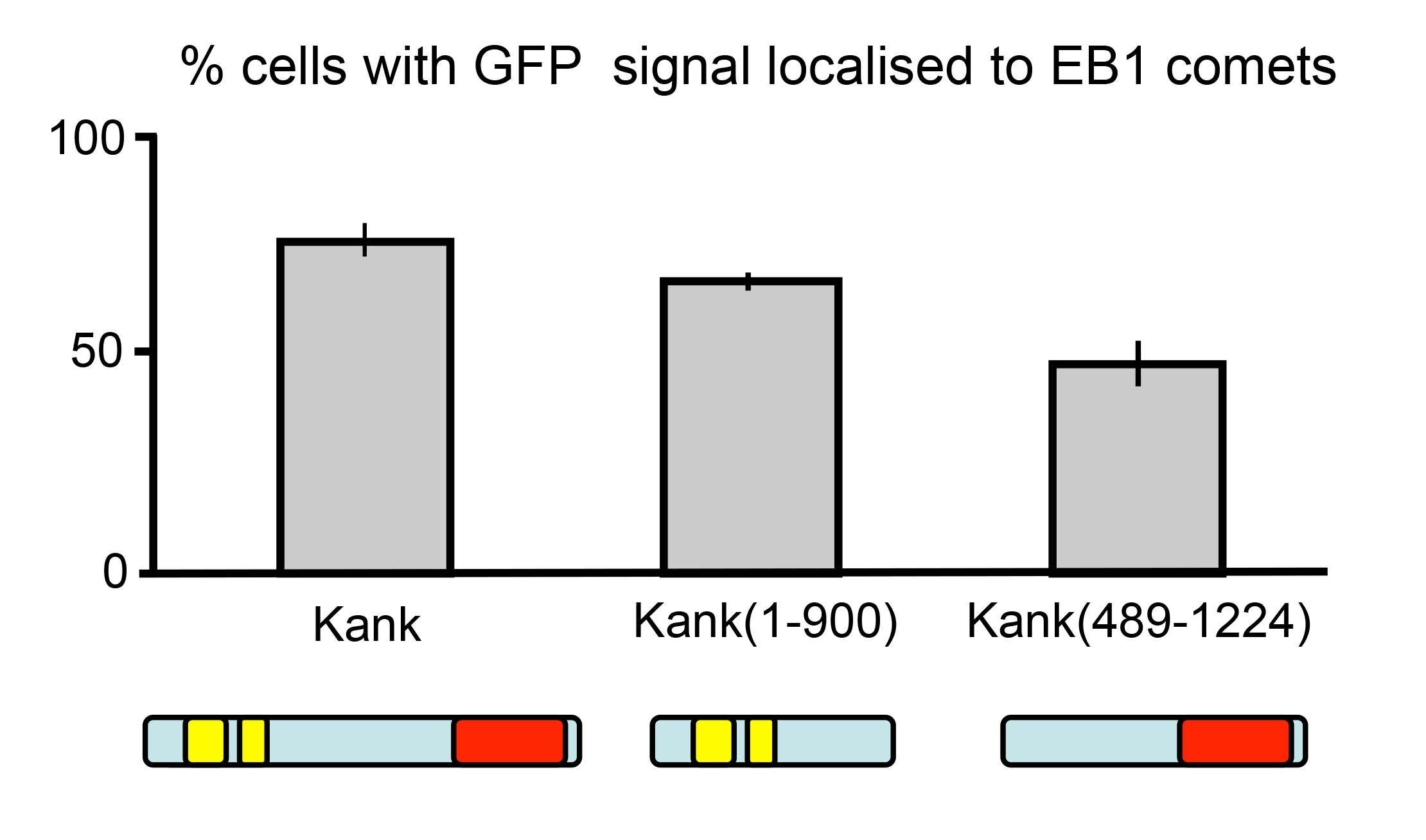

Supplement: Figure S1 — Kank(1–889) and Kank(489–1224), co-localise with EB1. S2 cells transfected with GFP-Kank(1–889) and GFP-Kank(489–1224) were co-stained for GFP and EB1. The number of cells with GFP localised to the majority of the EB1 comets (>50% estimate) was counted. For both truncations colocalisation of the GFP signal with EB1 was observed in the majority of observed cells. Error bars show the standard error of the mean. (TIF) [file pone.0106112.s001.tif]

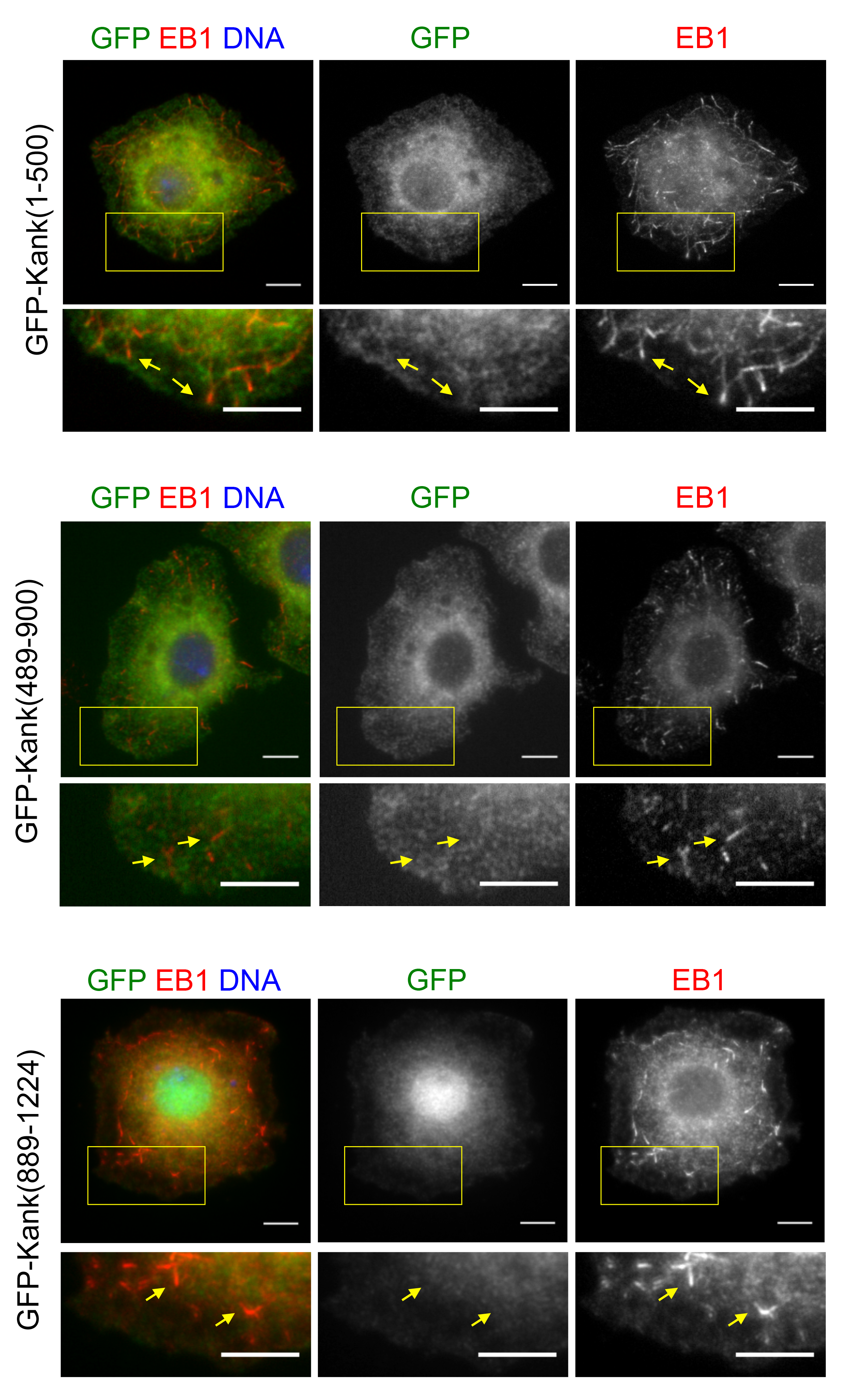

Supplement: Figure S2 — Kank(1–500), Kank(489–900) or Kank(889–1224) do not co-localise with EB1. S2 cells were transfected with Kank(1–500), Kank(489–900) or Kank(889–1224) and co-stained for GFP and EB1. These truncations showed diffuse localisation within the cytoplasm. In addition, Kank(1–500) and Kank(889–1224) localised to the nucleus. GFP signal was observed at the cell periphery for Kank(1–500) and Kank(489–900). Yellow boxes are areas magnified in images shown below. Scale bars = 5 µm. (TIF) [file pone.0106112.s002.tif]

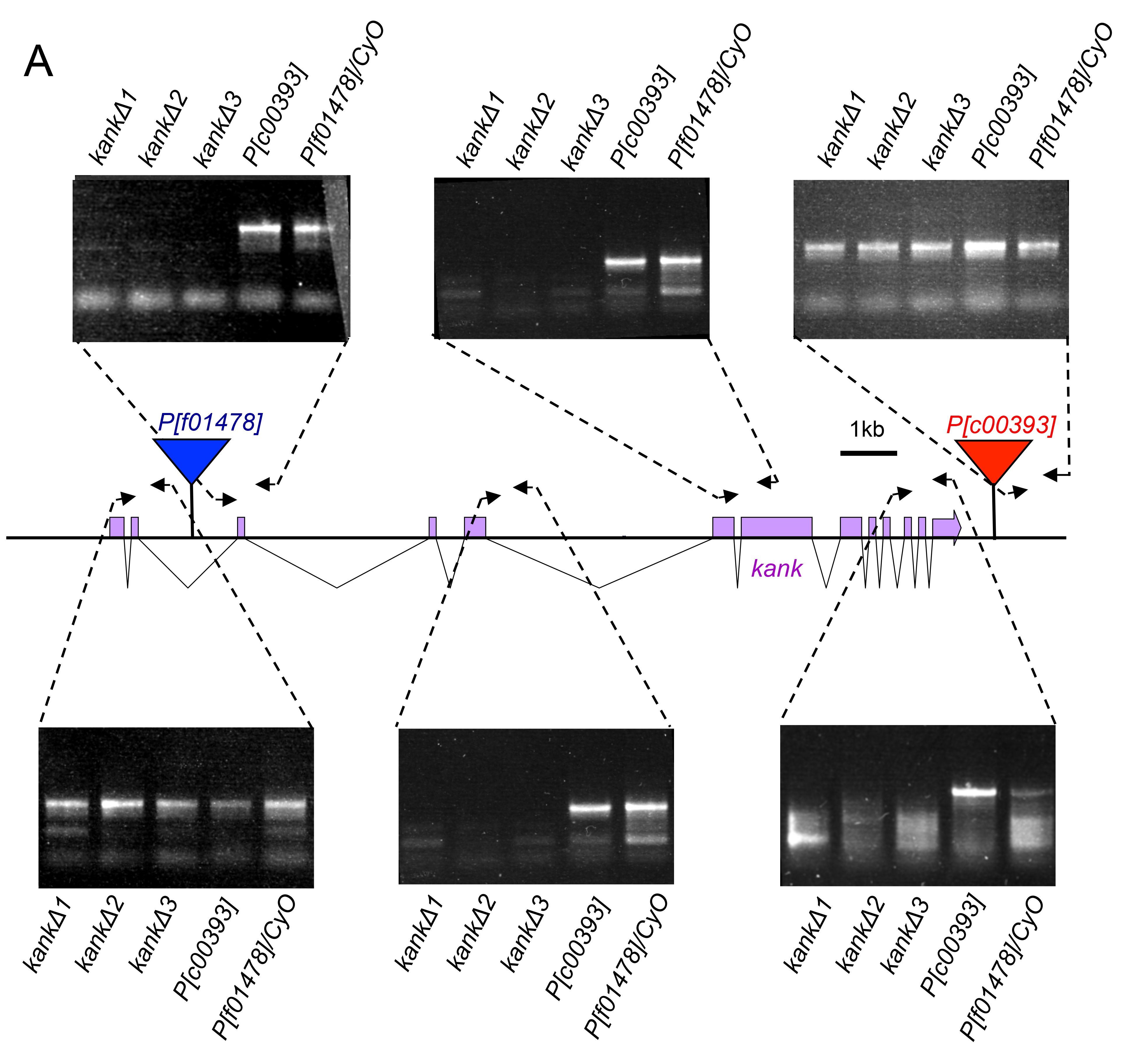

Supplement: Figure S3 — Deletion of Kank was confirmed by PCR. (A) PCR was carried out on genomic DNA of three kank deletion (Δ1, Δ2, Δ3) and two parental lines (P[c00393], P[f01478]) using designated primers (Table S1). (TIF) [file pone.0106112.s003.tif]

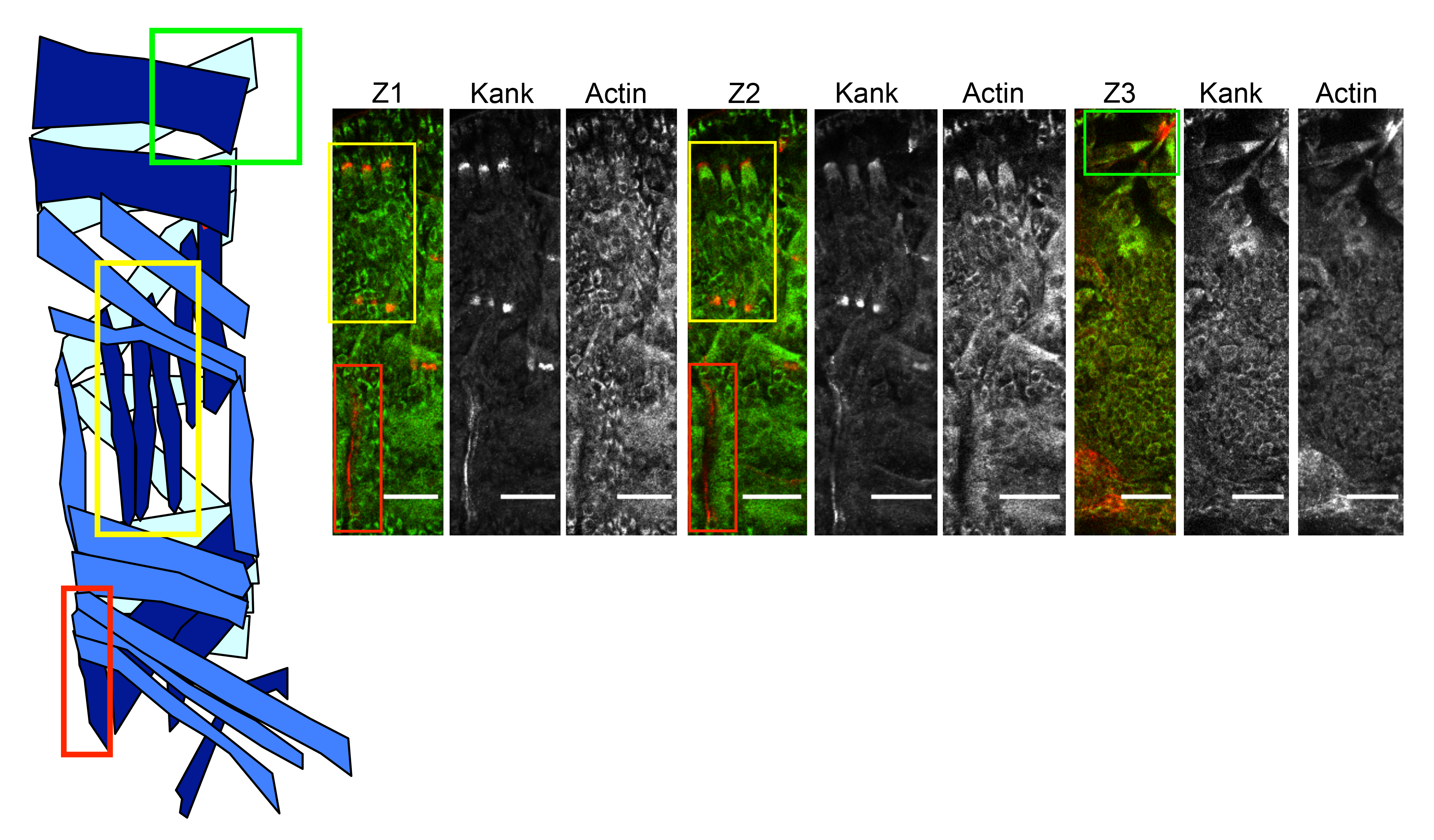

Supplement: Figure S4 — Kank localises to muscle tendon attachment sites. As actin staining is quite ubiquitous, Z sections of actin stained embryos were examined to visualise cells which resembled those muscle cells indicated in the schematic. The localisation of the Kank signal is observed at the sites of muscle-tendon attachment. Coloured boxes show the similarities between actin staining and the somatic muscle schematic. Scale bar = 25 µm. (TIF) [file pone.0106112.s004.tif]

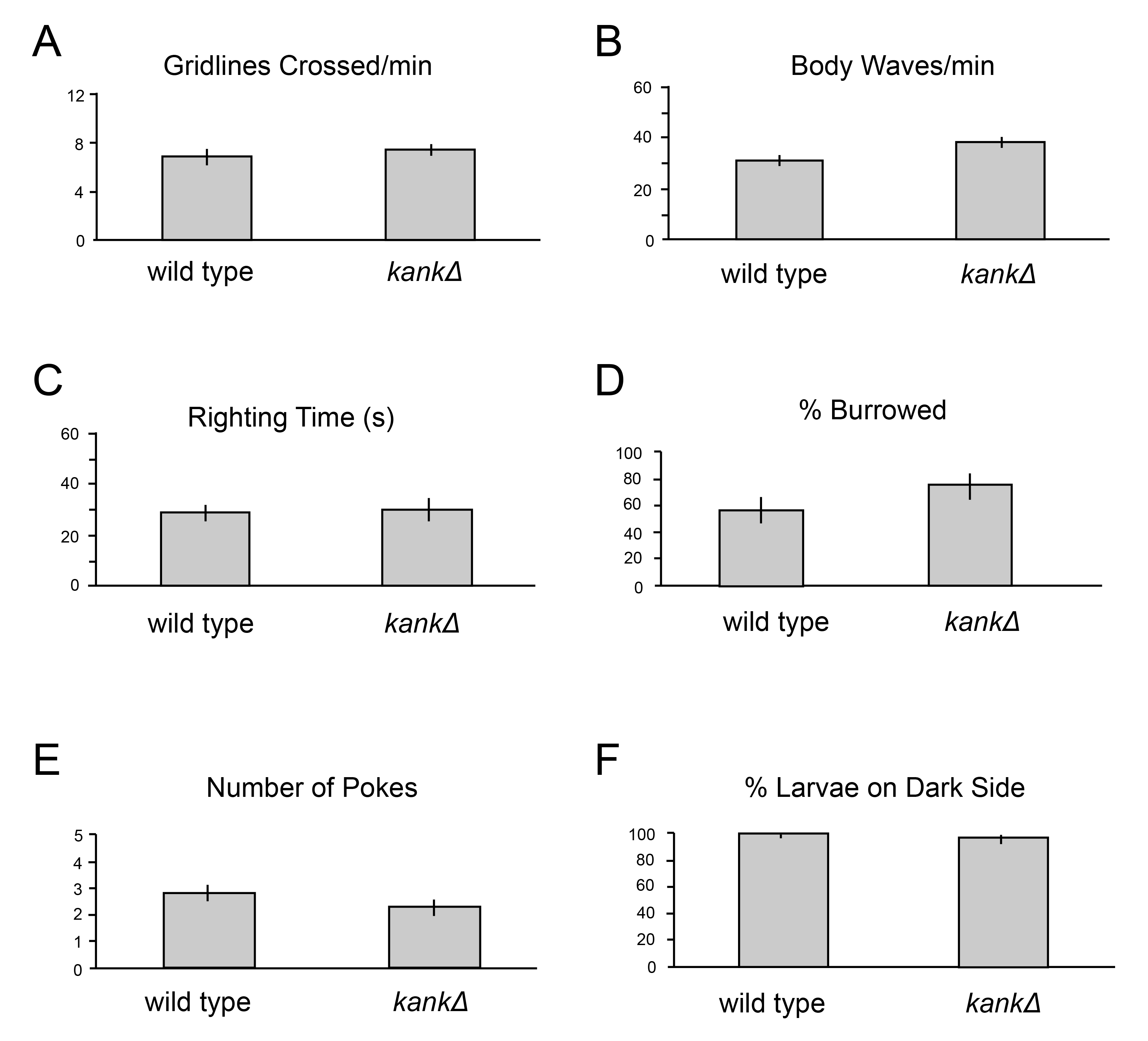

Supplement: Figure S5 — kankΔ larvae do not exhibit any motility or sensory defects. Unless otherwise stated, larvae examined were late 3rd instar (∼76 hours after hatching) and significance was determined by Student's t-test (A–C,E) or Fisher's exact chi-square test (D,F). Error bars show the standard error of the mean (A–C,E) or the 95% confidence interval (D,F). All assays were carried out at room temperature. (A) In the first assay the motility of larvae was examined (adapted from [43]) The number of gridlines passed by individual larva in 60 seconds was counted. The number of gridlines crossed by the kankΔ larvae was similar to that crossed by the wild type control (p>0.05). (B) In the second assay the overall coordination and motility of larvae was then examined by counting the number of full body motile waves (peristaltic waves) carried out by larvae in one minute (adapted from [44]). The peristaltic waves travelled the entire length of the larva in a coordinated fashion in both wild type and the mutant. The frequency of peristaltic waves was not significantly different between the kankΔ and wild type (p>0.05). (C) In the third assay, larvae were rolled from their ventral to their dorsal side while on an agarose plate (adapted from [45]). The time taken for them to right themselves was measured, with a maximum of 2 minutes allowed. The time taken by kankΔ and wild type was similar (∼30 seconds; p>0.05). (D) The fourth assay determined if larvae maintained burrowing ability. Foraging third instar larva were placed on top of food in a bottle which was then placed in the dark for 2 hours (adapted from [46]). After this time, the number of larvae remaining on the food was counted. No significant difference between the kankΔ and the wild-type larvae were observed (p>0.05). (E) Larvae were manually stimulated to elicit a nociceptive response (adapted from [47]). Third instar larvae was prodded by a blunt instrument at their abdominal segments and evading action was observed. No significan [file pone.0106112.s005.tif]
