# Supplementary material for: Kank Is an EB1 Interacting Protein that Localises to Muscle-Tendon Attachment Sites in Drosophila
Source: PLoS One. 2014 Sep 9;9(9):e106112. doi: 10.1371/journal.pone.0106112 (PMC4159139; doi:10.1371/journal.pone.0106112)
Supplement: Table S1 — Sequences of primers used to check for kank deletion in genomic DNA. (DOC) [file pone.0106112.s006.doc]

**Supplementary Table S1. Sequences of primers used to check for *kank* deletion in genomic DNA**

| **Names of primers** | **Primer sequences** |
| --- | --- |
| oSC119 | ATA CGC TGT GCA ACG AAC TTT |
| oSC120 | GAA CCA AGT GTG CAT GTT TCG TT |
| oSC125 | GTG GAG CAA CAT ATC GCC AGC GT |
| oSC126 | GTG GAG CAA CAT ATC GCC AGC GT |
| oSC129 | AGC CTA TTA GGA GGC GCG AT |
| oSC130 | GCA CGC AAT CTT CGA TGC GGA TAA |
| oSC131 | CAA GAA GGC CAT CGA GCG ACA T |
| oSC132 | CTC TGC CTA TTG GGC TAC CTG AA |
| oSC135 | CGG ACA GGT TGA CAC AGT ACT CCA GCA |
| oSC136 | GCC TGT GCA GCC AAC TAC TGT GAT |
